# Magnitude and precision of absolute blood volume estimated during hemodialysis

**DOI:** 10.1080/0886022X.2024.2377781

**Published:** 2024-08-15

**Authors:** Rammah Abohtyra, Tyrone Vincent, Daniel Schneditz

**Affiliations:** a Department of Electrical Engineering, The University of Texas Permian Basin, Midland, TX, USA; b Libyan Authority for Scientific Research, Tripoli, Libya; c Department of Electrical Engineering, Colorado School of Mines, Golden, CO, USA; d Division of Physiology and Pathophysiology, Otto Loewi Research Center for Vascular Biology, Immunology and Inflammation, Medical University of Graz, Graz, Austria

**Keywords:** Blood volume, hemodialysis, ultrafiltration, hematocrit, nonlinear estimation, unscented Kalman filter

## Abstract

**Background:** Management of body fluid volumes and adequate prescription of ultrafiltration (UF) remain key issues in the treatment of chronic kidney disease patients.**Objective:** This study aims to estimate the magnitude as well as the precision of absolute blood volume ($V_{b}$) modeled during regular hemodialysis (HD) using standard data available with modern dialysis machines.**Methods:** The estimation utilizes a two-compartment fluid model and a mathematical optimization technique to predict UF-induced changes in hematocrit measured by available on-line techniques. The method does not rely on a specific hematocrit sensor or a specific UF or volume infusion protocol and uses modeling and prediction tools to quantify the error in $V_{b}$ estimation.**Results:** The method was applied to 21 treatments (pre-UF body mass: 65.57±13.44 kg, UF-volume: 3.99±1.14 L) obtained in ten patients (4 female). Pre-HD $V_{b}$ was 5.4±0.53 L with an average coefficient of variation of 9.8% (range 1 to 22%). A significant moderate correlation was obtained when $V_{b}$ was compared to a different method applied to the same data set (*r* = 0.5). Specific blood volumes remained above the critical level of 65 mL/kg in 17 treatments (80.9%).**Conclusion:** The method offers the opportunity to detect critical blood volumes during HD and to judge the quality and reliability of that information based on the precision of the $V_{b}$ estimate.

## Graphical abstract

**Figure:**
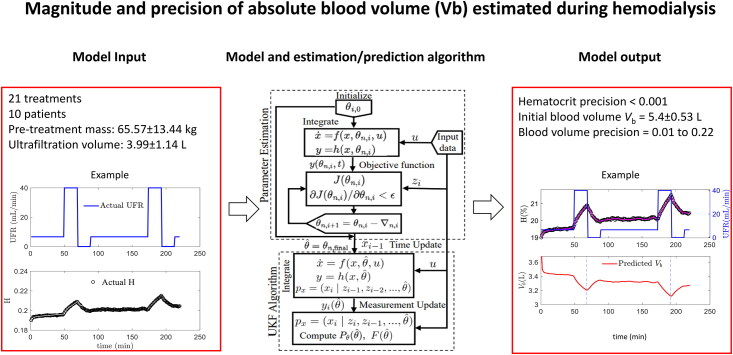

## 1. Introduction

Chronic kidney disease (CKD) is a major health concern, significantly decreasing the patient’s quality of life, causing a high mortality, and creating a substantial economic impact [1,2]. Most CKD patients receive an intermittent therapy of 3–4 hemodialysis sessions (each session is 2–5 h long) per week to support or to completely replace their lost kidney function. Apart from water-soluble metabolites removed by diffusion and adjustment of acid-base and electrolyte balance when blood is exposed to dialysate in the extracorporeal circulation, excess fluid accumulated in the typical 2-day interval throughout the body leading to an intradialytic body mass gain in the range of 2–4 kg has to be removed by ultrafiltration (UF) of blood to reestablish so-called dry body mass at the end of each single treatment session [3].

Intradialytic hypotension (IDH) is a common complication of hemodialysis and is associated with a high risk of cardiovascular morbidity and mortality [4–8]. The single most important factor for IDH is acute hypovolemia, either because of errors in prescribing the proper ultrafiltration volume as the true dry body mass is essentially unknown, or because prescribed ultrafiltration rates largely exceed the rates at which extravascular fluid is refilled into the vascular compartment. Depending on the patient’s hemostatic defense mechanisms to compensate for acute hypovolemia, IDH may then develop with all unwanted side effects. Therefore, knowing a patient’s blood and fluid volumes at the start of dialysis and during ultrafiltration could allow clinicians to avoid critical degrees of hypovolemia and to safely return CKD patients to their dry body mass [9,10].

Dry body mass, adequate ultrafiltration, and absolute blood volume have been of concern since the early days of dialysis. However, most standard measuring methods are not feasible in everyday clinical practice so that ultrafiltration of excess volume remains largely prescribed by clinical judgment only.

All current methods to measure absolute blood volume are based on some form of indicator dilution and require the precise administration and measurement of that indicator. The removal of excess fluid by ultrafiltration itself can also be seen as a form of indicator dilution, where fluid volume is removed (rather than added) and the indicator, in this case red blood cells, is therefore concentrated (rather than diluted). Several techniques for online measurement of blood components such as hematocrit, total protein or hemoglobin concentration are now available in clinical practice but so far, they are only used for estimating relative blood volume changes during hemodialysis and ultrafiltration.

It is the aim of this study to examine whether current methods measuring hematocrit, total protein or hemoglobin concentration can also be used to identify absolute blood volume without relying on specific ultrafiltration and infusion protocols. The approach suggested in this manuscript is based on fitting a blood volume model to blood concentration data recorded during a dialysis session in clinical routine. The identification of absolute blood volume for the treatment session is achieved by combining a modeling algorithm with a prediction algorithm using a modified two-compartment blood volume model to provide the magnitude as well as the precision of absolute blood volume.

## 2. Materials and methods

### 2.1. Mathematical model

**Figure 1.**
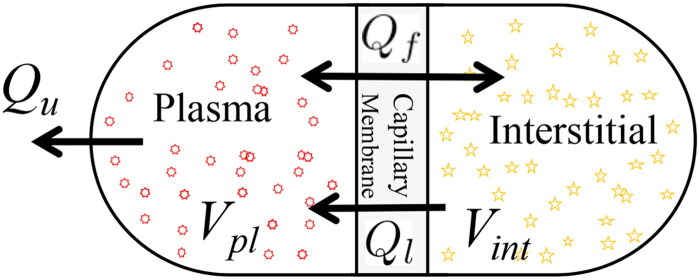
The two-compartment model consists of interstitial ($V_{\text{int}}$) and intravascular (plasma) fluid volumes ($V_{\text{pl}}$) separated by a microvascular membrane; arrows indicate microvascular ($Q_{f}$), lymph ($Q_{l}$), and ultrafiltration flows ($Q_{u}$).

The volume kinetics during ultrafiltration is described by a previously published two-compartment extracellular fluid volume model [11,12]. Some modifications such as a constant protein mass in each compartment and a lymphatic flow component were introduced to the model [11,13–16] (Figure 1).

The states of the model are given as the plasma volume, $V_{\text{pl}}$ (L), and the interstitial fluid volume, $V_{\text{int}}$ (L); while the input, $Q_{u}$, is the ultrafiltration rate (UFR) (L/min). The output is the whole body hematocrit (*H*), which is the volume fraction of all red blood cells ($V_{\text{rbc}}$, L) in total blood volume ($V_{b}=V_{\text{rbc}}+V_{\text{pl}}$), i.e., $H=V_{\text{rbc}}/V_{b}$.

The mathematical description of this model during dialysis is given by the nonlinear differential system:

(1a) $${\dot{V}}_{\text{pl}}=-Q_{f}(V_{\text{pl}},V_{\text{int}})+Q_{l}(V_{\text{int}})-Q_{u}$$

(1b) $${\dot{V}}_{\text{int}}=Q_{f}(V_{\text{pl}},V_{\text{int}})-Q_{l}(V_{\text{int}})$$

(1c) $$H=\frac{V_{\text{rbc}}}{V_{b}}$$

where $Q_{f}$ (L/min) represents the microvascular (shift/refill) filtration flow, in which $K_{f}$ (L/min.mmHg) is the transcapillary permeability coefficient, and $Q_{\text{ly}}$ (L/min) represents the lymphatic flow. Details of the model are explained in Appendix A.1.

Additionally, the absolute blood volume $V_{b}(t)$ is given as the sum of variable plasma volume $V_{\text{pl}}(t)$ and constant red blood cell volume $V_{\text{rbc}}$. Six parameters including initial conditions for plasma ($V_{\text{pl}}(0)$) and interstitial volumes ($V_{\text{int}}(0)$) are considered patient specific $\theta ={\left[V_{\text{pl}}(0),V_{\text{int}}(0),V_{\text{rbc}},K_{f},d_{1},d_{2}\right]}^{T}$, whereas the other parameters are assumed as nominal for all patients (Table 1).

**Table:** Glossary of terms.

Symbol	Generic term
*V*	Volume (L)
*v*	Specific blood volume (mL/kg)
*M*	Mass (kg)
*C*	Concentration (mg/L)
*Q*	Fluid flow rates (volume/time)
UFR, ( $Q_{u}$ )	Ultrafiltration rate (volume/time)
$V_{u}$	Ultrafiltration volume (L)
*t*	time (min)
$T_{s}$	Sampling time (min)
*E*	Expected value
*H*	Hematocrit
*pl*, *int*, *e*	subscript, referring to plasma, interstitial, extracellular
*b*, *rbc*, *f*	subscript, referring to blood, red blood cell, filtration
*l*, *c*, *b*	subscript, referring to lymph, capillary, blood
θ , $\hat{\theta }$	referring to parameters, estimated parameters
*g*, *h*, β	Constants
*p*	Hydrostatic pressure (mmHg)
π	Colloid-osmotic pressure (mmHg)

Kalman filtering (named after the Hungarian-American electrical engineer and mathematician Rudolf E. Kálmán, 1930–2016) is a mathematical algorithm widely used in signal processing, control systems, navigation and control. It is a filter incorporating internal feedback to estimate the system’s varying quantities (its state) from a series of noisy measurements. In the application here it uses the dynamic two-compartment fluid volume model described above, ultrafiltration as a known control input to that system, and multiple serial blood measurements to obtain an estimate of the system’s state (i.e. blood concentrations such as hematocrit) that is better than the estimate obtained by using only one measurement alone. The unscented Kalman filter (UKF) represents a variant accounting for the non-linearity of the dynamic model.

**Appendix Figure 1.**
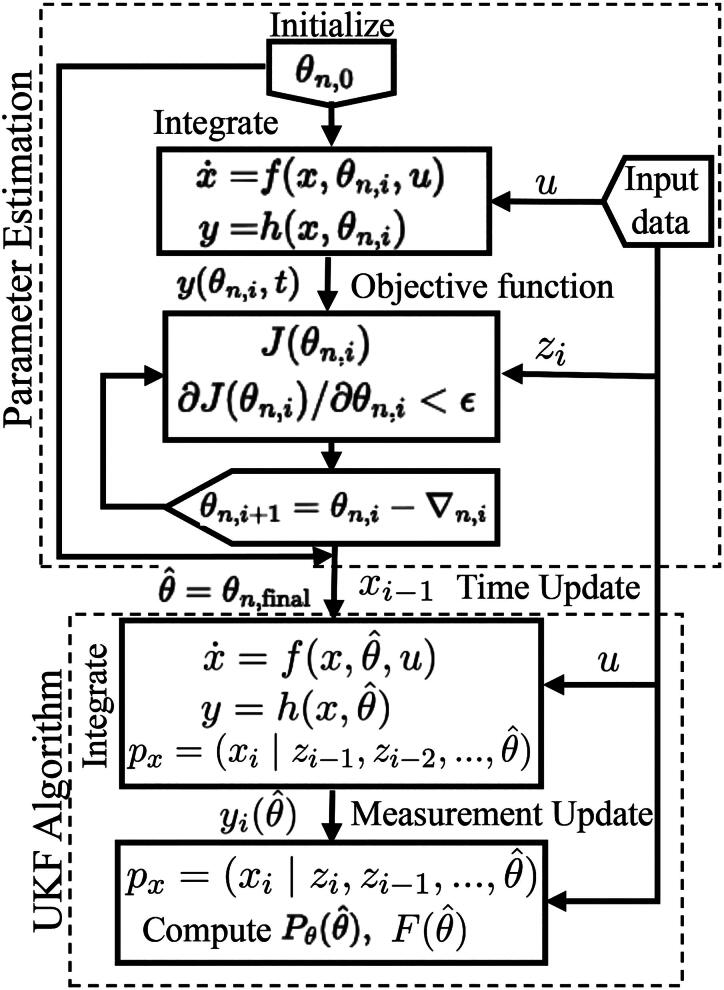
Modeling algorithm. The parameter estimation and unscented Kalman filter (UKF) prediction algorithms are independently performed. The estimated parameter $\hat{\theta }$ is an input to the UKF algorithm.

The parameter estimation and prediction algorithms for the model are summarized in Appendix Figure 1 and detailed in Appendix B.1, C.1, and D.1 [17].

### 2.2. Initial conditions

Initial conditions are based on physiologic considerations. Normal blood is about 70 mL/kg and scales with body mass [18]. The density of the whole body and of body fluid volumes is close to 1 kg/L so that the numerical value is maintained when masses are converted to volumes. The ratio of blood to extracellular volume is about 1/3 [19]. This ratio is maintained with moderate volume expansion [20]. Volume expansion was determined from the difference of pre- to post-treatment body mass. Interstitial protein concentration is about 1/3 of plasma protein concentration. The protein mass (M=C×V) in each of the compartments was assumed as constant.

- Initial blood volume $V_{b}(0)$ = 70 mL/kg × mass post + (mass pre - mass post)/3.
- Initial extracellular volume $V_{e}(0)$ = (3 × 70 mL/kg) × mass post + (mass pre - mass post).
- Initial plasma volume $V_{\text{pl}}(0)=V_{b}(0)\times (1-H(0))$.
- Initial interstitial volume $V_{\text{int}}(0)=V_{e}(0)-V_{\text{pl}}(0)$.
- Initial microvascular coefficient $K_{f}=8.1\times 10^{-5}$ (L/(min.mmHg.kg)) × mass post [11].
- Initial plasma protein mass $M_{\text{pl}}(0)=C_{\text{pl}}(0)\times V_{\text{pl}}(0)$.
- Initial interstitial protein mass $M_{\text{int}}(0)=(C_{\text{pl}}(0)/3)\times V_{\text{int}}(0)$.
- Red blood cell volume $V_{\text{rbc}}=V_{b}(0)\times H(0)$.

These relationships create a patient specific initial condition $\theta_{0}=[V_{\text{pl}}(0),V_{\text{int}}(0),V_{\text{rbc}},K_{f},d_{1},d_{2}]$. Upper and lower boundaries of $\theta_{0}$ within plausible physiological ranges (±50% of $\theta_{0}$) centered at $\theta_{0}$ create randomly initial conditions $\theta_{n,0}$ for each patient. To address the sensitivity to initial conditions (initial guess for θ), the uncertainty and uniqueness of the optimal solution is examined using randomly selected sets of initial parameters $\theta_{n,0}$ (positive and uniform distribution) within bounded intervals. The output provides a distribution of optimal solutions represented by Kernel Probability Density (KPD) functions estimated over the random initial values $\theta_{n,0}$ and describes the quality of a given estimation.

### 2.3. Clinical data

The method was tested in highly dynamic hematocrit data collected in 21 studies obtained in ten patients using specific ultrafiltration profiles [21]. The variability induced by the ultrafiltration profiles is especially useful to test the performance of new method. The data are also useful as the blood volume identified by a different, independent method at two time points ($v_{1}$, $v_{2}$) serves to examine the quality of the blood volume estimate obtained in the current study. The experimental study was approved by the Internal Review Board of the Medical School of the Karl-Franzens University in Graz without restriction to use anonymized data in follow-up analyses. All study participants provided informed consent. Patient information such as sex, body mass at treatment end, ultrafiltration volume, and initial plasma protein concentration were also available and used for parameter estimation (Table 2).

**Table 1.** Nominal model parameters.

Constants	value
*a*	0.006
α	−198
γ	−45
*g*	0.045
η	0.767
β	0.045
$p_{0}$ (mmHg)	13.128
$V_{p,\text{eu}}$ (L)	3
$V_{\text{int},\text{eu}}$ (L)	11
$k_{c1}$	0.21
$k_{c2}$	0.0016
$k_{c3}$	$9\times 10^{-6}$

A 60 min training phase of combined ultrafiltration rate input and hematocrit data was used to train the model and to estimate the parameter θ, and then the rest of the data was used to validate the results and test the quality of the model.

### 2.4. Statistics

Data are presented as average values ± standard deviation (SD) or coefficients of variation (CV) unless otherwise indicated. Variables obtained by different methods were compared by linear regression and Bland-Altman analyses.

The quality of the output was quantified by a normalized mean square error (MSE) between modeled and measured variables:

(2) $$\text{MSE}(H,\hat{H})=\frac{1}{\bar{H}}\underset{k}{\sum }{\left(H(k)-\hat{H}(t_{k}�\hat{\theta })\right)}^{2}$$

where $\hat{H}(t_{k}�\hat{\theta })$ is the modeled hematocrit at time interval *k* of the measured hematocrit taken from the model’s output trajectory generated by integrating the model forward with the estimated parameters, and $\bar{H}$ is an average value of the measured hematocrit.

## 3. Results

**Figure 2.**
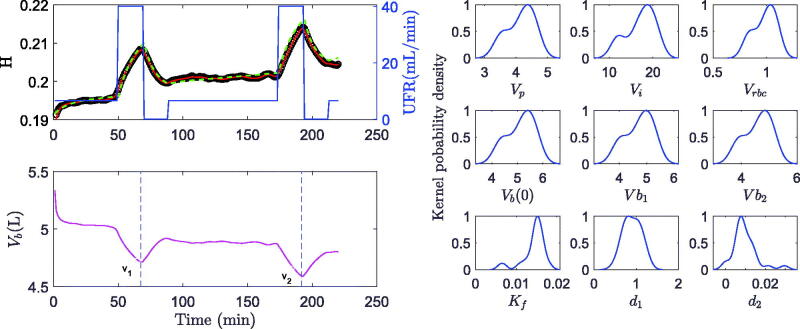
Modeling and prediction. Left, top panel: Modeled hematocrit (green dashed trajectory) compared to predicted hematocrit (red trajectory) using UKF versus actual hematocrit data (black circles) (study #3) in response to variable ultrafiltration rate (UFR, blue line). Left, bottom panel: Predicted blood volume (magenta) and time points (dashed blue lines) of blood volumes estimated at the end of ultrafiltration steps ($v_{1}$, $v_{2}$). right panels: Kernel probability density functions for various parameters over the random initial solution $\theta_{n,0}$.

The time course of hematocrit measured in 21 hemodialysis treatments was successfully modeled by the new algorithm and provided plausible model parameters. A representative example of measured, modeled, and predicted hematocrit is shown in Figure 2.

The up and down in hematocrit is untypical when compared to data obtained during routine hemodialysis and can be explained by the ultrafiltration profile used in this study. As expected, hematocrit increased when fluid volume was removed from plasma volume by ultrafiltration, and dropped when ultrafiltration was stopped and fluid was refilled from the expanded interstitial space. Overall, there was an increase in hematocrit with ongoing ultrafiltration, and the increase was smooth with a constant ultrafiltration rate. Figure 2 shows an example of a well-modeled hematocrit. The hematocrit prediction using the UKF approach is only unremarkably better than the modeled hematocrit. The KPD functions show unimodal and close to normal distributions around the estimated parameter maxima.

**Figure 3.**
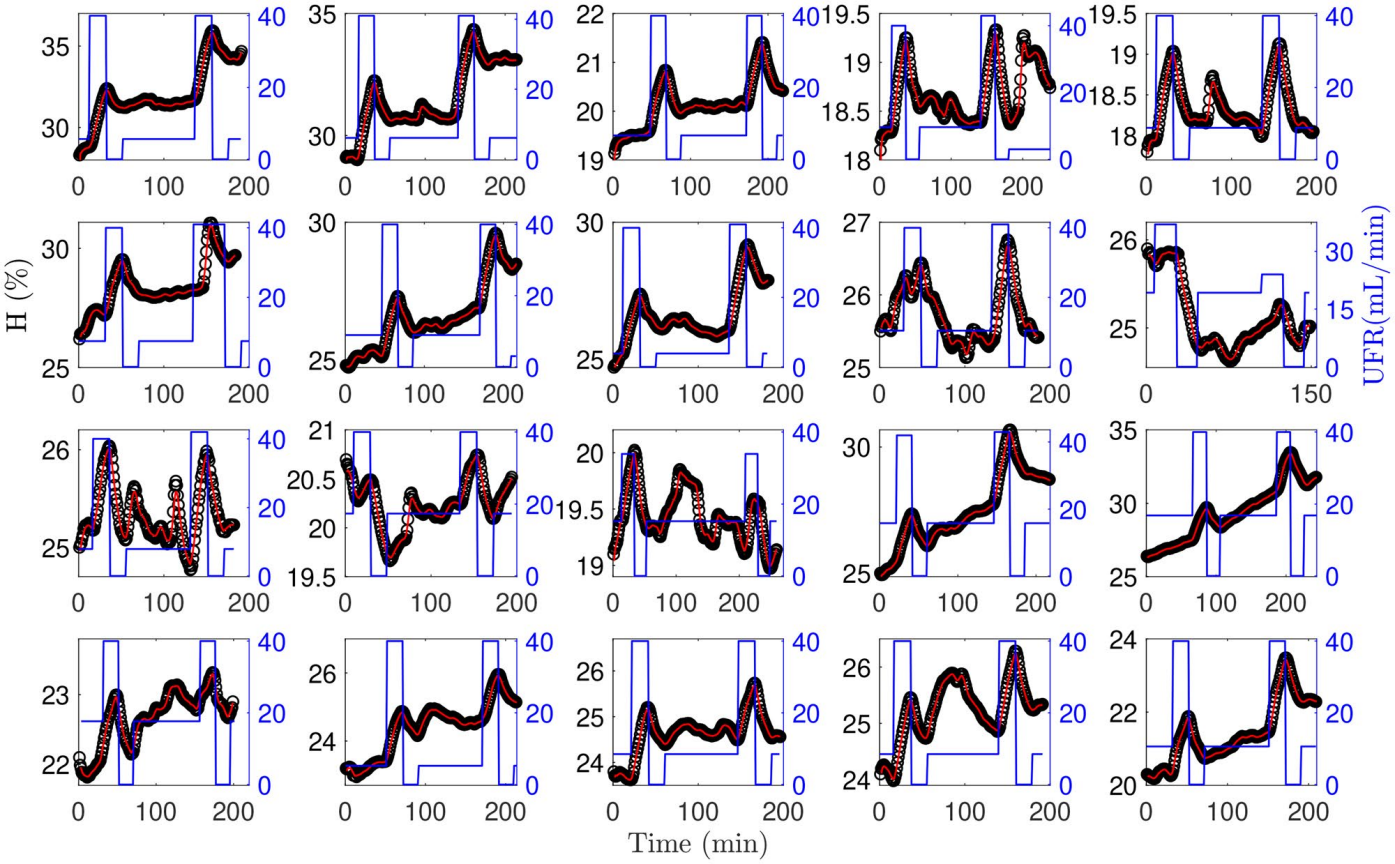
Prediction and measurement. Comparison of predicted (red lines) and measured (black circles) hematocrit in studies #1 to #20 using ultrafiltration rate profiles (blue lines) (study #21 is omitted because of limited space).

Figure 3 shows a summary plot of individual measurements. The corresponding model parameters of all studies are listed in Tables 3 and 4. Notice that all parameters of interest are obtained with distinct standard deviations. The average CV for estimated blood and plasma volumes was in the range of 10% (Table 3). Specific blood volumes were plausible and in the range of 78.7 and 75.9 mL/kg at the end of the high ultrafiltration rate pulse (Table 3). Specific volumes below the critical level of 65 mL/kg were observed in four studies done in three patients, in one of those (study #16), absolute blood volume was already below the critical threshold at treatment start.

**Table 2.** Patient and treatment information. Abbreviations: male (m), female (f), pre-dialysis body mass (M pre), post-dialysis body mass (M post), ultrafiltration volume ( $V_{u}$ ), treatment time, initial hematocrit H(0) , initial plasma protein concentration $C_{p}(0)$ , standard deviation (SD).

		M pre	M post	$V_{u}$	time	H(0)	$C_{p}(0)$
Study	Sex	(kg)	(kg)	(L)	(min)	(%)	(g/L)
1	m	76.2	74.0	2.5	240	28.3	66
2	m	76.5	74.0	2.8	270	29.1	70.4
3	f	62.8	60.0	3.3	240	19.0	58.7
4	f	63.2	60.0	3.2	240	18.1	65.2
5	f	62.5	60.0	3.0	240	17.8	63.9
6	f	52.9	50.0	3.7	360	26.2	64.3
7	f	53.3	50.0	4.1	360	24.8	63.6
8	f	52.5	50.0	2.7	360	24.7	65.3
9	m	67.8	64.5	3.6	270	25.5	72.5
10	m	68.5	64.8	4.3	240	25.9	74.9
11	m	67.1	64.5	2.9	240	25.0	70.8
12	m	62.3	58.0	4.9	270	20.7	68.8
13	m	61.2	58.0	3.8	240	19.1	63.4
14	f	56.4	52.8	4.7	270	25.0	64.2
15	f	57.0	52.5	4.8	270	26.4	63.1
16	m	117.3	110.0	7.6	420	22.1	67.6
17	m	67.1	64.0	3.7	480	23.2	65.9
18	m	68.4	64.0	5.0	480	23.8	69.3
19	m	68.3	64.0	4.9	480	24.1	67.8
20	f	53.5	50.5	3.3	240	20.3	70.1
21	m	62.2	58.5	5.0	300	21.6	68.4
mean		65.57	62.10	3.99	310	23.37	66.87
SD		13.44	12.76	1.14	85.52	1.65	3.66

**Table 3.** Identified volumes. Abbreviations: Estimation results (mean ± SD), initial blood volume $V_{b}(0)$ , minimum specific blood volume $v_{1}$ and $v_{2}$ at UF steps, initial plasma and interstitial volumes $V_{\text{pl}}(0)$ , $V_{\text{int}}(0)$ , mean square error (MSE), and *specific blood volume ≤ 65 mL/kg.

	$V_{b}(0)$	$v_{1}$	$v_{2}$	$V_{\text{pl}}$	$V_{\text{int}}$	MSE $\times 10^{-3}$
Study	(L)	(ml/kg)	(ml/kg)	(L)	(L)	(-)
1*	6 ± 0.25	69.4	62.6	4.3 ± 0.18	15 ± 1.18	0.31
2*	5.4 ± 0.07	63.9	60.0	3.9 ± 0.05	14.7 ± 1.2	0.26
3	5.1 ± 0.50	75.1	73.1	4.1 ± 0.4	17.1 ± 3.13	0.0038
4	5.4 ± 0.52	81.3	81.0	4.4 ± 0.42	20.5 ± 3.84	0.069
5	5.5 ± 0.28	82.6	82.2	4.5 ± 0.23	20.7 ± 2.02	0.069
6	5.1 ± 0.35	85.5	85.1	3.8 ± 0.26	14.5 ± 2.8	0.063
7	5.2 ± 0.85	88.1	81.3	3.9 ± 0.43	16.2 ± 2.65	0.26
8	5.7 ± 0.85	98.3	92.5	4.3 ± 0.64	16.5 ± 4.24	0.057
9	5.2 ± 0.63	73.7	72.9	3.9 ± 0.47	16.7 ± 4.25	0.02
10	6.5 ± 0.40	95.1	97.4	4.8 ± 0.30	27.2 ± 2.99	0.063
11	5.9 ± 0.21	85.3	85.5	4.4 ± 0.15	24.6 ± 1.66	0.048
12	5.8 ± 0.16	95.4	94.3	4.6 ± 0.13	27.9 ± 1.7	0.013
13	5.7 ± 0.28	89.2	90.9	4.6 ± 0.23	22.2 ± 2.11	0.32
14	5.5 ± 0.84	88.5	79.1	4.1 ± 0.64	16.4 ± 4.12	0.05
15	5.1 ± 0.78	76.1	67.5	3.6 ± 0.58	13.9 ± 3.76	0.01
16*	5.1 ± 0.64	42.1	41.6	4.0 ± 0.49	17.5 ± 4.27	0.087
17	5.3 ± 0.73	72.8	69.7	4.0 ± 0.56	15.7 ± 3.46	0.049
18	5.6 ± 0.91	77.3	75.7	4.3 ± 0.69	18.3 ± 4.97	0.066
19	5.2 ± 1.14	72.4	70.1	3.9 ± 0.87	15.8 ± 6.19	0.13
20	4.7 ± 0.35	80.4	74.9	3.7 ± 0.28	14.8 ± 2.26	0.081
21*	4.3 ± 0.58	60.9	56.7	3.4 ± 0.45	13.3 ± 2.46	0.2
Average	5.4 ±0.53	78.7	75.9	4.12 ±0.4	18.1 ± 3.1	0.12

**Table 4.** Identified model parameters (mean ± standard deviation). Abbreviations: filtration coefficient ( $K_{f}$ ), hydrostatic pressure parameters ( $d_{1}$ and $d_{2}$ ), and red blood cell volume ( $V_{\text{rbc}}$ ).

	$K_{f}$			
Study	(mL/(min × mmHg))	$d_{1}$ (-)	$d_{2}\times 10^{-3}$ (-)	$V_{\text{rbc}}$ (L)
1	5.3 ± 0.6	0.8 ± 0.44	4.1 ± 3	1.7 ± 0.07
2	5.9 ± 0.4	0.9 ± 0.34	6.1 ± 4	1.6 ± 0.02
3	14.3 ± 3	0.9 ± 0.19	9.7 ± 6	0.9 ± 0.09
4	24.6 ± 3	0.3 ± 0.26	5 ± 1	0.9 ± 0.09
5	20.9 ± 3	0.5 ± 0.14	1.2 ± 2	0.9 ± 0.05
6	8.6 ± 3	1.2 ± 0.17	10 ± 5	1.3 ± 0.09
7	9.9 ± 3	1.1 ± 0.24	9.6 ± 5	1.3 ± 0.14
8	12.7 ± 3	0.6 ± 0.33	2.3 ± 3	1.4 ± 0.21
9	84.1 ± 3	0.9 ± 0.39	5.6 ± 4	1.3 ± 0.16
10	17.1 ± 3	0.33 ± 0.29	6 ± 1	1.7 ± 0.10
11	34.3 ± 3	0.9 ± 0.19	7.8 ± 3	1.5 ± 0.05
12	25.3 ± 3	0.2 ± 0.19	0.5 ± 2	1.2 ± 0.03
13	25.6 ± 3	0.4 ± 0.20	0.4 ± 1	1.0 ± 0.05
14	9.3 ± 3	0.6 ± 0.32	2.8 ± 3	1.4 ± 0.21
15	6.5 ± 3	0.9 ± 0.38	5.4 ± 3	1.3 ± 0.21
16	19.3 ± 3	0.6 ± 0.24	2.3 ± 2	1.1 ± 0.14
17	11.0 ± 3	0.9 ± 0.22	7.3 ± 6	1.2 ± 0.17
18	13.5 ± 3	0.5 ± 0.45	1.5 ± 2	1.3 ± 0.22
19	17.6 ± 3	0.3 ± 0.29	1.9 ± 2	1.3 ± 0.27
20	10.7 ± 3	0.9 ± 0.23	11.6 ± 6	0.9 ± 0.07
21	4.8 ± 3	1.1 ± 0.26	23.1 ± 1.5	0.9 ± 0.13
**Average**	18.2 ± 4.5	0.7 ±0.25	5.4 ± 3.7	1.3 ±0.12

**Figure 4.**
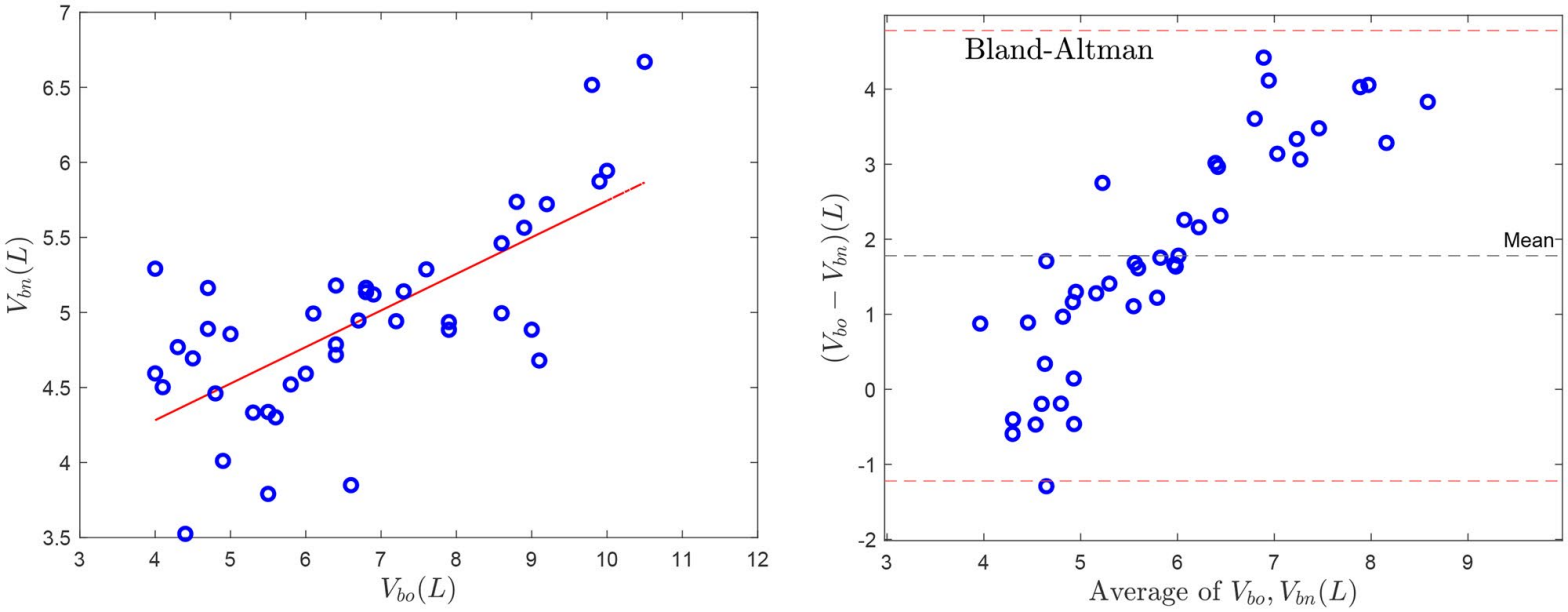
Comparison of methods. Left panel: Identity plot and linear regression ($V_{\text{bn}}=0.244\times V_{\text{bo}}+3.308$, r=0.5, red line) of blood volumes determined by previous ($V_{\text{bo}}$) and new methods ($V_{\text{bn}}$). right panel: Bland-Altman analysis of difference of estimated volumes ($V_{\text{bn}}$–$V_{\text{bo}}$) versus average of both volumes, mean of differences (black dashed line) and mean ±2 standard deviations, red dashed lines.

A comparison of volumes obtained by the new approach with results from the previous analysis is provided in Figure 4. The correlation (*r*) is significant, albeit moderate (0.5 ≤r≤0.85). The old method overestimates blood volumes in the high-volume range when compared to the new method.

**Figure 5.**
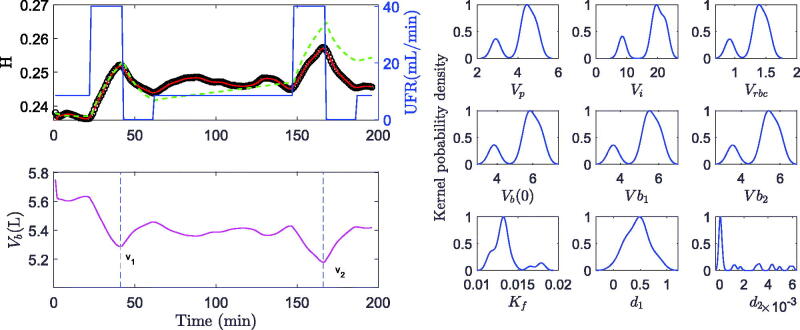
Modeling and prediction. Left, top panel: Poorly modeled hematocrit (green dashed trajectory) compared to predicted hematocrit (red trajectory) using UKF versus actual hematocrit (black circles) (study #18) in response to variable ultrafiltration rate (UFR, blue line). Left, bottom panel: Predicted blood volume (magenta) and time points (dashed blue lines) of blood volumes estimated at the end of ultrafiltration steps ($v_{1}$, $v_{2}$). right panels: Kernel probbility density functions for various parameters over the random initial solution $\theta_{n,0}$.

An example of a poorly-modeled hematocrit course and the associated kernel probability distributions are presented in Figure 5. Contrary to the poor modeling indicated by an increasing deviation between modeled and measured hematocrit values for advanced treatment phases, a close match with negligible errors between measured and predicted hematocrit was obtained using the UKF approach throughout the whole observation phase. The associated KPD distribution in blood and fluid volumes is wider and bimodal. Similar distributions and variations in blood and fluid volume estimations were identified in six out of 21 treatments (28%).

## 4. Discussion

A new parameter and state estimation approach to identify blood volume in CKD patients is presented using experimental hematocrit and ultrafiltration data continuously recorded during hemodialysis. The model is based on a modified two-compartment volume kinetic model, anthropometric patient information, as well as specific treatment information. A short training phase is utilized to estimate the model parameters and initial conditions and then the rest of the data are used for model validation. Using this method, the whole time course of hematocrit is predicted with a single set of estimated parameters, ultrafiltration rates, and the prediction (UKF) algorithm.

The data show increased dynamics with two distinct hematocrit peaks within the first and the last hours of the 4 h observation phase (Figure 3) because ultrafiltration was varied between very high and very low rates for a different purpose. Such an ultrafiltration profile is atypical in normal dialysis prescription as ultrafiltration is usually delivered at a constant rate causing a much smoother hematocrit time course [22]. However, data recorded with variable ultrafiltration rates stimulating a characteristic system response have been used since the beginning of blood volume modeling [12]. In general, they are better suited to test algorithms as parameter estimation and hematocrit prediction is more challenging with increased dynamics. The data are also characterized by low hematocrit values in the range of 25% as the measurements were done before erythropoiesis stimulating agents became available which typically increased hematocrit to about 35% [23].

In all treatments the predicted hematocrit closely fits the actual hematocrit (Figures 2, 3, 5) whereas in six treatments the model alone (without UKF) failed to adequately describe the whole *H* profile. A closer look at one of these treatments (Figure 5) reveals a discrepancy between modeled and measured hematocrit values starting in the period between ultrafiltration pulses. In this phase patients were allowed to sit up, change their body position, or to have a small snack, all of which affects the driving forces for microvascular fluid shifts as well as the distribution of red blood cells within the cardiovascular space [24,25]. As a result, measured hematocrit varies independently of actual ultrafiltration rate. Since the current model does not account for effects of orthostasis and food intake, the effect of unknown inputs cannot adequately be modeled. Similar difficulties have previously been addressed by introducing an active control of lymphatic refilling [12].

The hematocrit prediction using the UKF algorithm, however, also very closely matches the actual measurements in this case. In other words, even if the model is inadequate to describe the dynamics of the variable of interest, the UKF approach accounts for the error in model structure and still provides a very close prediction.

This, however, introduces a different problem. Usually, the quality of a model is judged by the goodness of fit between modeled and measured data. The close fit between predicted (not modeled) and measured data using the UKF approach might be mistaken for a high quality of the modeled parameters and could be misleading. It is therefore important to also examine the precision of identified model parameters. In this study the KPD function for $V_{b}(0)$ is wide and bimodal (Figure 5) and the resulting CV for initial blood volume is 16.3% and higher compared to the average CV of 9.8% for the whole group. Therefore, the volume estimate in this specific treatment is less precise despite very close hematocrit prediction using the UKF approach.

The value of the new approach is not so much in providing a precise prediction of the hematocrit, but in providing a measure for the precision of identified parameters. A precise prediction of hematocrit is not required because this variable is available and continuously measured by various on-line techniques. The practical interest of modeling is with precise identification of system parameters and variables which have clinical relevance but which cannot directly be measured. Parameters such as absolute blood volume must be inferred from modeling, usually by some type of indicator dilution. Different methods have been proposed and applied in clinical practice, but the precision of parameter identification is rarely addressed. Without information on precision, parameter identification is incomplete.

In this study the absolute blood volume was identified with an average CV of 9.8% (range 1 to 22%) while a precision of better than 5% would be desirable. An improved precision could be obtained by modifying and expanding the model, for example with regard to hemodynamic effects of ultrafiltration [26]. This, however requires more patient and treatment information and model assumptions, and it remains to be decided, whether such an expansion is feasible.

The method presented in this study can be used to identify critical specific blood volumes below which patients are more likely to experience intradialytic morbid events [27]. Specific blood volumes fell below the critical threshold of 65 mL/kg in three studies and were below that value from the beginning of treatment in one study. None of the patients became symptomatic during any of the treatments. However, this threshold (65 mL/kg) could be different when using highly dynamic ultrafiltration profiles in an experimental situation reducing the sensitivity of the threshold. The general applicability of this threshold therefore remains to be confirmed in larger clinical studies.

**Figure 6.**
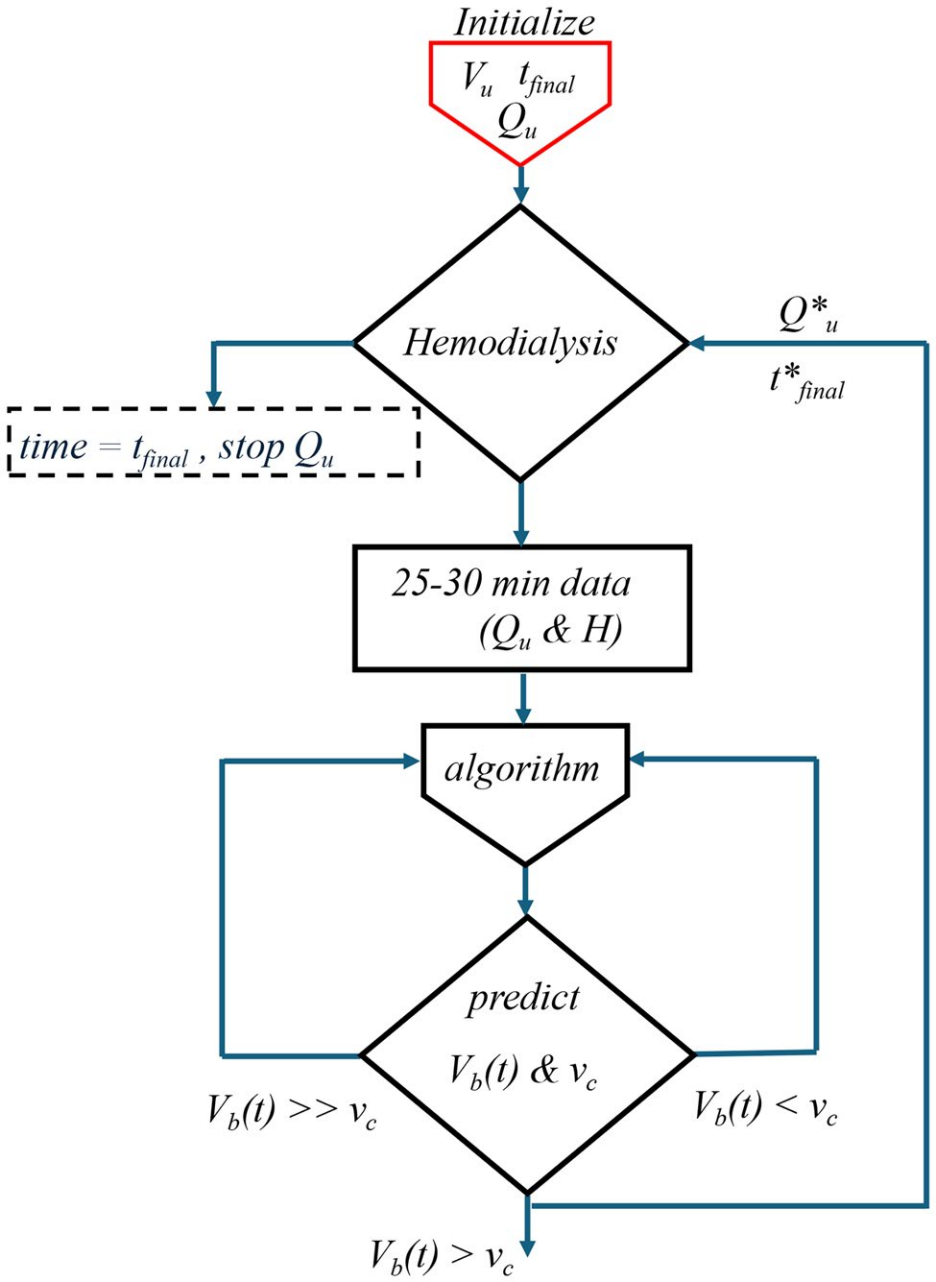
IDH prevention algorithm. For $V_{u}$ with initial $Q_{u}$ and $t_{\text{final}}$, 25–30 min is used to predict $V_{b}(t)$ (L) and critical value ($v_{c}$ (L)) with three scenarios to prevent IDH 1) if $V(t)<v_{c}$ reduce $Q_{u}$ and increase $t_{\text{final}}$, 2) if $V(t)\gg v_{c}$, increase $Q_{u}$ and decrease $t_{\text{final}}$, and 3) $Q_{u}^{*}$ and $t_{\text{final}}^{*}$ are used to remove $V_{u}$ if $V(t)>v_{c}$.

Once the validity of an accepted blood volume threshold is confirmed in independent studies, the proposed method to predict the absolute blood volume could assist in the prevention of IDH. Using the new approach with a valid threshold, one could predict the risk of critical specific blood volumes ahead of time. This could then be used to adjust the ultrafiltration rate to avoid critical blood volumes (Figure 6). This method could also be coupled to available feedback- control systems using any sensor continuously measuring concentration or relative blood volume data [28–31].

It is a limitation of the study that system parameters were not validated by accepted and independent reference methods. Absolute blood volumes were, however, compared to blood volumes identified by a different method applied to the same data set. This comparison provides a significant albeit moderate correlation, where volumes identified by the previous method appear to be increasingly overestimated at increasing blood volume range (Figure 4). Extracellular volume modeled from whole-body bioimpedance spectroscopy could have been used to compare the sum of plasma and interstitial volumes. It also remains to be tested whether the new approach provides precise and non-trivial volume estimates in treatments with constant ultrafiltration rates.

## 5. Conclusions

The new estimation approach offers a practical method for estimating absolute blood volume in everyday dialysis using continuous hematocrit and ultrafiltration data. The absolute blood volume estimated at the beginning and during every dialysis session offers the opportunity to improve fluid management in CKD patients. This method also can be used to determine critical specific blood volumes during single dialysis treatments and to detect low absolute blood volumes ahead of time.

## Appendix A

### A.1 Blood volume model

The microvascular (shift/refill) filtration and lymphatic flows are given by:

(A1) $$Q_{f}(V_{\text{pl}},V_{\text{int}})=K_{f}(\delta_{\text{pl}}-\delta_{\pi })$$

(A2) $$\begin{array}{cc} Q_{l}(V_{\text{int}})= & g\text{tanh}(h\pi_{\text{int}})+\beta \end{array}$$

where $\delta_{\text{pl}}$ and $\delta_{\pi }$ represent the hydrostatic and colloid osmotic pressure gradients, respectively:

(A3) $$\delta_{\text{pl}}(t)=(p_{c}(t)-\pi_{\text{int}}(t))$$

(A4) $$\begin{array}{cc} \delta_{\pi }(t)= & \left(\pi_{\text{pl}}-\pi_{\text{int}}(t)\right) \end{array}$$

and

(A5) $$p_{c}(t)=d_{1}{\left(100\left(\frac{V_{\text{rbc}}+V_{\text{pl}}(t)}{V_{\text{rbc}}+V_{p,\text{eu}}}\right)+r\right)}^{d_{2}+p_{0}}$$

(A6) $$p_{\text{int}}(t)\;=\left(100\left(\frac{aV_{\text{int}}(t)}{V_{\text{int},\text{eu}}}\right)\;+\frac{\alpha }{\left(100\frac{V_{\text{int}}}{V_{\text{int},\text{eu}}}+\gamma \right)}\right)$$

(A7) $$\pi_{\text{pl}}(t)=\left(\frac{k_{c1}M_{\text{pl}}}{V_{\text{pl}}(t)}+\frac{k_{c2}M_{\text{pl}}^{2}}{V_{\text{pl}}^{2}(t)}+\frac{k_{c3}M_{\text{pl}}^{3}}{V_{\text{pl}}^{3}(t)}\right)$$

(A8) $$\pi_{\text{int}}(t)=\left(\frac{k_{c1}M_{\text{int}}}{V_{\text{int}}(t)}+\frac{k_{c2}M_{\text{int}}^{2}}{V_{\text{int}}^{2}(t)}+\frac{k_{c3}M_{\text{int}}^{3}}{V_{\text{int}}^{3}(t)}\right)$$

where $p_{c},$ $p_{\text{int}}$, $\pi_{\text{pl}}$, $\pi_{\text{int}}$ refer to the hydrostatic capillary, interstitial, plasma colloid osmotic, and interstitial colloid osmotic pressures, ­respectively, defined in [11–13], where *g*, *h*, and β are constants, and where $V_{p,\text{eu}}$, $V_{\text{int},\text{eu}}$ denote euvolemic plasma and interstitial volumes for a 70 kg patient, respectively; $p_{0}$ is an offset pressure; $M_{\text{pl}}$ is plasma protein mass; $M_{\text{int}}$ is interstitial protein mass, and *a*, α, γ, $d_{1}$, $d_{2}$, *r*, $k_{c1}$, $k_{c2}$ and $k_{c3}$ are nominal parameters of model (1) (Table 1). The equations of these components, nominal parameters, and constants are provided in [13].

## Appendix B

### B.1. Parameter estimation

The estimation approach utilizes a general nonlinear model of a continuous-time differential equation described by a state transition ($f_{s}$) and observation function ($h_{o}$):

(B1a) $$\dot{x}=f_{s}(x,\theta ,u,t)$$

(B1b) $$\begin{array}{cc} y= & h_{o}(x,\theta ,t)+w \end{array}$$

where $x\in R^{n}$ denotes the state of the system; *u* denotes the input to the system; and $\theta \in R^{m}$ contains the model parameters whose values (or some of them) are estimated from data; *y* is the model output; *t* is the time $t_{0}\leq t\leq t_{\text{final}}$ between initial, $t_{0}$, and final time, $t_{\text{final}}$; and *w* represents measurement noise.

The algorithm optimizes the parameters θ by minimizing the sum of the square errors between the measured ($H_{i}$) and the model’s $\left(y(\theta ,t_{i})\right)$ (1) in response to the ultrafiltration (*u*) as an input to the model, where $t_{i}$ indicates the time of *H* measurements (i=1,…,N). The objective function is given by

(B2) $$\underset{\theta }{\text{min}}(J(\theta ))=\underset{\theta }{\text{min}}\sum_{i=1}^{N}(H_{i}-y(\theta ,t_{i}){)}^{2}$$

where $H_{i}$ and $y(\theta ,t_{i})$ refer to observed measurements and output trajectory of the nonlinear model, respectively. The optimal solution of (B2) is denoted by $\hat{\theta }$. The estimation algorithm consists of two nested loops (Appendix Figure 1): the outer one loops over a random set of initial conditions $\theta_{n,0}$ [33], and the inner loop is based on the Levenberg-Marquardt method where the value $\theta_{n}$ is updated on the *i*th cycle by $\theta_{n,i+1}=\theta_{n,i}-\nabla_{n,i}$ where $\nabla_{n,i}$ uses the steepest descent method.

Note that the estimated parameter $\hat{\theta }$ is an input to the Unscented Kalman Filter (UKF) prediction algorithm.

## Appendix C

### C.1. Prediction

The UKF [34,35] is integrated with the nonlinear model and data to compute the estimation error using the Fisher information matrix, to update states (blood and fluid volumes), and to predict the measured hematocrit.

To estimate the parameter uncertainty and make predictions of state trajectories, using UKF, we introduce the model uncertainty (*n*) into (B1a)

(C1) $$\dot{x}=f(x,\hat{\theta },u,t)+n$$

and assume that the distribution of model uncertainty (*n*) and measurement noise (*w*) in (B1b) is piece-wise constant over intervals of $T_{s}$ ($T_{s}$ is a sampling time), the magnitude for the interval *k* (*k* is an index) is a Gaussian random vector with zero mean given by

(C2a) $$n_{k}∼N(0,\Sigma )$$

(C2b) $$v_{k}∼N(0,R)$$

and where N represents the notation for normal Gaussian distribution; Σ and *R* are the covariance of model uncertainty and measurement noise, respectively. Consequently, we assume *x* is Gaussian. Since *y* is conditionally affected by the state *x*, we also assume that *y* is Gaussian. Thus, the conditional state and output density functions are Gaussian:

(C3a) $$p_{x}(x_{i}⏧x_{i-1},u_{i-1},\hat{\theta })=N(x_{i},\Sigma )$$

(C3b) $$\begin{array}{cc} p_{y}(y_{i}⏧x_{i},\hat{\theta })= & N(h(x_{i},\hat{\theta }),R) \end{array}$$

where $x_{i}$ is the solution to (B1) with n=0, initial condition $x_{i-1}$ and input $u_{i-1}$.

As illustrated in Appendix Figure 1, the prediction algorithm uses the UKF with the estimated parameter $\hat{\theta }$ and the nonlinear model (B1) to compute the Fisher information matrix $F(\hat{\theta })$, update states, and ­predict the output of this model. Here, we provide iterative steps for updating the UKF, utilizing observations $z_{0}$, $z_{1},\ldots ,z_{N}$ at a sampling time $T_{s}$, and an input *u*, where *u* is held as a piecewise constant during the interval $(i-1)T_{s}\leq t\leq iT_{s}$:

- $p_{x}(x_{i-1}⏧z_{i-1},z_{i-2},\ldots ,\hat{\theta })$ represents the previous estimated state.
- $p_{x}(x_{i}⏧z_{i-1},z_{i-2},\ldots ,\hat{\theta })$ represents the prediction update step (time update), obtained by integrating model (B1) between time $t=(i-1)T_{s}$ to $t=iT_{s},$ with initial conditions $x((i-1)T_{s})=E[x_{i-1}⏧z_{i-1,}z_{i-2},\ldots ,\hat{\theta }]$.
- $y(\hat{\theta })=E[h(x_{i},\hat{\theta })⏧z_{i-1},z_{i-2},\ldots ,\hat{\theta }]$ represents the prediction step of a measurement $z_{i}.$
- $p_{x}(x_{i}⏧z_{i},z_{i-1},z_{i-2},\ldots ,\hat{\theta })$ represents the measurement update step of the UKF.

Algorithm 1 below presents the parameter estimation (A) and prediction (UKF) algorithm (B).

Algorithm 1: The Parameter and Prediction Algorithms **Table:**

A. Parameter estimation algorithm
1: **For over** n=1:M
2: Initialize $\theta_{n,0}$
3: ** While *i*** (check $⏧\partial J(\theta_{n,i})/\partial \theta_{n,i}⏧<\epsilon )$
4: Numerically integrate $\dot{x}=f(\theta_{n,i},u),y(\theta_{n,i})=h(x,\theta_{n,i})$
5: Compute $e_{n,i}(\theta_{n,i})=y(\theta_{n,i},t_{i})-z(t_{i})$ , $J(\theta_{n,i})=\sum_{k=0}^{N}e^{2}(\theta_{n,i})\;$
6. $\theta_{n,i+1}=\theta_{n,i}-\nabla_{n,i}$ ( $\nabla_{n,i}$ steepest descent)
7: **End While**
8: $\hat{\theta }=\theta_{n,\text{final}}$
9. **End For**
B. State estimation and prediction (**UKF**) algorithm
1: **For over** i=1:N
2: Numerically integrate $\dot{x}=f(\hat{\theta },u),y(\hat{\theta })=h(x,\hat{\theta })$
from time $t=(i-1)T_{s}$ to $t=iT_{s},$ with initial condition.
$x((i-1)T_{s})=E[x_{i-1}⏧y_{i-1,}y_{i-2},\ldots ,\hat{\theta }]$ .
3: Update $p_{x}(x_{i}⏧y_{i-1},y_{i-2},\ldots ,\hat{\theta })$ (Time update).
4: Predict $y_{i}$ as $\hat{y}(\hat{\theta })=E[h(x_{i},\hat{\theta })⏧y_{i-1},y_{i-2},\ldots ,\hat{\theta }].$
5: Update $p_{x}(x_{i}⏧y_{i},y_{i-1},y_{i-2},\ldots ,\hat{\theta })$ (Measurement update).
6. **End for**
7: Compute $p_{y⏧\hat{\theta }}(y_{i}⏧\hat{\theta }),$ $P_{\theta }(\hat{\theta })$ , and Fisher matrix $F(\hat{\theta })$ .

## Appendix D

### D.1. Sensitivity analysis

We use the Fisher information matrix [36] to quantify the precision of the estimation based on model uncertainty and measurement noise. This analysis uses the following assumptions.

- The distribution of y can be approximated by a Gaussian distribution.
- The experimental uncertainty is captured by model (B1)
- The covariance of the prediction error is independent of θ.
- The UKF is approximately optimal, so that the prediction errors are uncorrelated.
- The parameter estimates are unbiased.

Theorem D.1. *Under the assumptions listed above, the covariance of the parameter estimates (*$P_{\theta }$*) is bounded below by*

(D1) $$P_{\theta }\geq {\left(\sum_{i=0}^{N-1}\frac{\text{dy}{\left(\hat{\theta }\right)}^{T}}{d\hat{\theta }}S_{i}^{-1}\frac{\text{dy}(\hat{\theta })}{d\hat{\theta }}\right)}^{-1}$$

*where* $S_{i}$ *is the covariance of the prediction error calculated using the UKF.* The proof can be found elsewhere [17].

## References

[1] US Department of Health and Human Services. Centers for Disease Control and Prevention. Chronic Kidney Disease in the United States; 2021. [cited on Aug 19, 2022]. Available from: https://www.niddk.nih.gov/health-information/health-statistics/kidney-disease.

[2] Shlipak MG, Tummalapalli SL, Boulware LE, et al. The case for early identification and intervention of chronic kidney disease: conclusions from a kidney disease: improving global outcomes (KDIGO) controversies conference. Kidney Int. 2021;99(1):34–47. doi: 10.1016/j.kint.2020.10.012. 33127436

[3] Dasgupta I, Farrington K, Davies SJ, et al. UK National Survey of practice patterns of fluid volume management in haemodialysis patients: a need for evidence. Blood Purif. 2016;41(4):324–331. doi: 10.1159/000444246. 26863433

[4] Kanbay M, Ertuglu LA, Afsar B, et al. An update review of intradialytic hypotension: concept, risk factors, clinical implications and management. Clin Kidney J. 2020;13(6):981–993. doi: 10.1093/ckj/sfaa078. 33391741 PMC7769545

[5] Sars B, van der Sande FM, Kooman JP. Intradialytic Hypotension: mechanisms and Outcome. Blood Purif. 2020;49(1–2):158–167. doi: 10.1159/000503776. 31851975 PMC7114908

[6] Palmer BF, Henrich WL. Recent advances in the prevention and management of intradialytic hypotension. J Am Soc Nephrol. 2008;19(1):8–11. doi: 10.1681/ASN.2007091006. 18178796

[7] Daugirdas JT. Pathophysiology of dialysis hypotension: an update. Am J Kidney Dis. 2001;38(Suppl 4):S11–S17. doi: 10.1053/ajkd.2001.28090. 11602456

[8] Yildiz AB, Vehbi S, Covic A, et al. An update review on hemodynamic instability in renal replacement therapy patients. Int Urol Nephrol. 2023;55(4):929–942. doi: 10.1007/s11255-022-03389-w. 36308664

[9] Thijssen S, Kappel F, Kotanko P. Absolute blood volume in hemodialysis patients: why is it relevant, and how to measure it. Blood Purif. 2013;35(1–3):63–71. doi: 10.1159/000345484. 23343548

[10] Dekker M, Konings C, Canaud B, et al. Pre-dialysis fluid status, pre-dialysis systolic blood pressure and outcome in prevalent haemodialysis patients: results of an international cohort Study on behalf of the MONDO initiative. Nephrol Dial Transplant. 2018;33(11):2027–2034. doi: 10.1093/ndt/gfy095. 29718469

[11] Schneditz D, Roob J, Oswald M, et al. Nature and rate of vascular refilling during hemodialysis and ultrafiltration. Kidney Int. 1992;42(6):1425–1433. doi: 10.1038/ki.1992.437. 1474776

[12] Chamney P, Johner C, Aldridge C, et al. Fluid balance modelling in patients with kidney failure. J Med Eng Technol. 1999;23(2):45–52. doi: 10.1080/030919099294276. 10356673

[13] Abohtyra R, Chait Y, Germain MJ, et al. Individualization of ultrafiltration in hemodialysis. IEEE Trans Biomed Eng. 2019;66(8):2174–2181. doi: 10.1109/TBME.2018.2884931. 30530307 PMC6691497

[14] Abohtyra R, Hollot C, Germain MG, et al. Personalized ultrafiltration profiles to minimize intradialytic hypotension in end-stage renal disease. 2018 IEEE Conference on Decision and Control (CDC); Miami, Florida, USA. IEEE; 2018. p. 309–314. doi: 10.1109/CDC.2018.8619152.

[15] Abohtyra RM, Hollot C, Horowitz J, et al. Designing robust ultrafiltration rate profiles based on identifying fluid volume model parameters during hemodialysis. Dynamic Systems and Control Conference; Vol. 58271; Tysons, Virginia, USA. American Society of Mechanical Engineers; 2017. p. V001T08A006. doi: 10.1115/DSCC2017-5341.

[16] Fuertinger DH, Kappel F, Meyring-Wösten A, et al. A physiologically based model of vascular refilling during ultrafiltration in hemodialysis. J Theor Biol. 2016;390:146–155. doi: 10.1016/j.jtbi.2015.11.012. 26643943

[17] Abohtyra R, Vincent T, Schneditz D. Nonlinear parameter and state estimation approach for intradialytic measurement of absolute blood volume. 2023; Available from: https://www.biorxiv.org/content/10.1101/2023.08.12.553092v1.

[18] Prothero JW. The Design of Mammals. Cambridge (UK): Cambridge University Press; 2015.

[19] Guyton AC, Hall JE. Textbook of medical physiology. (9th edn). Philadelphia Pennsylvania: WB Saunders Company; 1996.

[20] Kron S, Schneditz D, Kron J. The blood to extracellular volume relationship is stable and in the physiologic range in chronic haemodialysis patients. Nephrol Dial Transplant. 2022;37(10):2034–2036. doi: 10.1093/ndt/gfac151. 35425989

[21] Schneditz D, Roob JM, Vaclavik M, et al. Noninvasive measurement of blood volume in hemodialysis patients. J Am Soc Nephrol. 1996;7(8):1241–1244. doi: 10.1681/ASN.V781241. 8866419

[22] Lopot F, Kotyk P. Computational analysis of blood volume dynamics during hemodialysis. Int J Artif Organs. 1997;20(2):91–95. doi: 10.1177/039139889702000207. 9093886

[23] Fogh-Andersen N, Eidemak I, Løkkegaard H, et al. Changes in blood and plasma volume during treatment with recombinant human erythropoietin. Scand J Clin Lab Invest Suppl. 1993;214(sup214):61–65. doi: 10.3109/00365519309090680. 8332853

[24] Shibagaki Y, Takaichi K. Significant reduction of the large-vessel blood volume by food intake during hemodialysis. Clin Nephrol. 1998;49(1):49–54. 9491287

[25] Sivalingam M, Banerjee A, Nevett G, et al. Haemodynamic effects of food intake during haemodialysis. Blood Purif. 2008;26(2):157–162. doi: 10.1159/000114094. 18230971

[26] Cavani S, Cavalcanti S, Avanzolini G. Model based sensitivity analysis of arterial pressure response to hemodialysis induced hypovolemia. Asaio J. 2001;47(4):377–388. doi: 10.1097/00002480-200107000-00016. 11482490

[27] Kron S, Schneditz D, Leimbach T, et al. Determination of the critical absolute blood volume for intradialytic morbid events. Hemodial Int. 2016;20(2):321–326. doi: 10.1111/hdi.12375. 26467262

[28] Kron S, Schneditz D, Leimbach T, et al. Feedback control of absolute blood volume: a new technical approach in hemodialysis. Hemodial Int. 2020;24(3):344–350. doi: 10.1111/hdi.12826. 32115891

[29] Paolini F, Mancini E, Bosetto A, et al. Hemoscan^™^: a ­dialysis machine-integrated blood volume monitor. Int J Artif Organs. 1995;18(9):487–494. doi: 10.1177/039139889501800902. 8582763

[30] Steuer RR. A new optical technique for monitoring ­hematocrit and circulating blood volume: its application in renal dialysis. Dial Transplant. 1993;22:260–265.

[31] Schneditz D, Pogglitsch H, Horina J, et al. A blood protein monitor for the continuous measurement of blood volume changes during hemodialysis. Kidney Int. 1990;38(2):342–346. doi: 10.1038/ki.1990.207. 2205752

[32] Abohtyra R, Vincent T, Schneditz D. New approach for intradialytic estimation of absolute blood volume during. J Am Soc Nephrol. 2023;34(11S):158–158. ­abstract). doi: 10.1681/ASN.20233411S1158c.

[33] Abohtyra RM, Chan CL, Albers DJ, et al. Inferring insulin secretion rate from sparse patient glucose and insulin measures. Front Physiol. 2022;13:893862. doi: 10.3389/fphys.2022.893862. 35991187 PMC9384214

[34] Julier SJ, Uhlmann JK. Unscented filtering and nonlinear estimation. Proc IEEE. 2004;92(3):401–422. doi: 10.1109/JPROC.2003.823141.

[35] Attarian A, Batzel JJ, Matzuka B, et al. Application of the unscented Kalman filtering to Parameter Estimation. In: Batzel JJ, Bachar M, Kappel F, editors. Mathematical modeling and validation in physiology: applications to the cardiovascular and respiratory systems. Heidelberg Germany: Springer Verlag; 2013. p. 75–88.

[36] Van Trees HL. Detection, Estimation, and Modulation Theory, part I: detection, Estimation, and Linear Modulation Theory. Hoboken, NJ: John Wiley & Sons; 2004.

